# Meta-analysis of acupuncture combined therapies for amblyopia: efficacy and safety insights

**DOI:** 10.3389/fmed.2025.1584296

**Published:** 2025-06-10

**Authors:** Kang Tan, Yunfeng Yu, Pei Liu, Pengfei Jiang, Yi Liu, Bidan Lou, Qinghua Peng

**Affiliations:** ^1^School of Traditional Chinese Medicine, Hunan University of Chinese Medicine, Changsha, China; ^2^Acupuncture-Tuina-Rehabilitation Department, The First Hospital of Hunan University of Chinese Medicine, Changsha, China; ^3^Quzhou Hospital Ophthalmology Center, Zhejiang Medical and Health University, Quzhou, China

**Keywords:** acupuncture, amblyopia, meta-analysis, systematic review, efficacy, safety

## Abstract

**Background:**

Acupuncture, a traditional Chinese medicine therapy, is widely used for the management of amblyopia. This study aimed to perform a meta-analysis of the efficacy and safety of acupuncture combined with conventional treatments for amblyopia.

**Methods:**

We searched eight databases for relevant studies published before December 31, 2024. Subsequently, the retrieved literature underwent a rigorous screening process based on pre-established inclusion and exclusion criteria. Thereafter, the basic information, outcome data, and risk of bias of the included studies were statistically analyzed. RevMan5.3 was employed to perform meta-analysis, sensitivity analysis, and assessment of publication bias. Additionally, GRADEpro3.6 was utilized to assess the quality of evidence for the outcomes.

**Results:**

Ten trials involving 918 eyes were included. The meta-analysis demonstrated that, compared with to the conventional treatment, the acupuncture combined with conventional treatment significantly improved the clinical efficacy rate (relative risk [RR] 1.31, 95% confidence interval [CI] 1.21–1.43, *P* < 0.00001, GRADE: low), the best-corrected visual acuity (BCVA) [mean difference (MD) 0.08, 95% CI 0.01–0.15, *P* = 0.03, GRADE: very low], and the amplitude of pattern visual evoked potential (P-VEP) P_100_ wave (MD 3.24, 95% CI 1.42–5.05, *P* = 0.0005, GRADE: low), while reduced the stereoacuity (MD −3.59, 95% CI −5.97 to 1.20, *P* = 0.003, GRADE: very low) and the latency of P-VEP P_100_ wave (MD −7.44, 95% CI −11.71 to −3.18, *P* = 0.0006, GRADE: very low). However, acupuncture may increase the adverse reaction rate (RR 5.57, 95% CI 1.01–30.84, *P* = 0.05, GRADE: low). Funnel plots indicated no publication bias in the clinical efficacy rate, latency of P-VEP P_100_ wave, amplitude of P-VEP P_100_ wave, or adverse events. Nevertheless, potential publication bias was detected for BCVA and stereoacuity.

**Conclusion:**

Compared to conventional treatment, acupuncture combined with conventional treatment effectively improved visual acuity in amblyopia patients, although it may increase the risk of adverse events. Considering that these adverse events are mild, acupuncture still has the potential to serve as a complementary treatment for amblyopia. However, these findings need to be further validated through large-scale and high-quality studies.

**Systematic review registration:**

https://www.crd.york.ac.uk/PROSPERO/view/CRD420251063432, identifier CRD420251063432.

## 1 Introduction

Amblyopia, also known as “lazy eye,” is a neurodevelopmental disorder that frequently affects children. It is characterized by reduced best-corrected visual acuity (BCVA) in one or both eyes compared to normal visual acuity for the corresponding age, despite the absence of any discernible organic ocular pathology (1). Global epidemiological data reveal a 1%–5% prevalence among children, with male predominance (male: female ratio ≈ 1.5:1) (2). Amblyopia materializes during a pivotal developmental epoch, presenting with visual impairments in the affected eye, sensory deficits in both the amblyopic and fellow eyes, and compromised binocular functionality (3). These deficits precipitate a decline in reading proficiency, visual-motor coordination, self-perception, and quality of life, ultimately exerting enduring detrimental effects on academic performance, social adaptation, and psychological health (4). Currently, the therapeutic methods for amblyopia include refractive correction, patching therapy, penalization therapy, visual training, and pharmacological and surgical interventions (5). However, the protracted treatment associated with refractive correction and patching therapy often causes discomfort and resistance in patients, and their efficacy is lacking in certain cases of severe and older amblyopia (6, 7). Penalization therapy exhibits considerable inter-individual variability in effectiveness, and prolonged penalization may precipitate a decline in visual acuity in the fellow eye (8). The outcomes of visual training are contingent on the intensity and duration of the regimen, making its efficacy inconsistent (9). The efficacies of pharmacological and surgical treatments are subject to individual variability, and their long-term effectiveness and safety require further investigation (10, 11). Consequently, although these modalities lead to a certain degree of improvement in patients’ vision, they are encumbered by numerous limitations.

Acupuncture, a traditional Chinese medicine therapy, has been employed as an adjunctive treatment for a spectrum of ophthalmological conditions, including myopia, dry eye, and glaucoma (12–14). The mechanism of action involves stimulating specific acupoints to enhance ocular microcirculation, thereby achieving therapeutic efficacy against eye diseases (15, 16). In recent years, there has been burgeoning interest in the role of acupuncture as a complementary treatment for amblyopia (17–19). Wang et al. (19) reported that acupuncture combined with conventional treatment significantly improved the corrected visual acuity and optic nerve conduction in children with anisometropic amblyopia. Cui et al. (18) found that acupuncture combined with conventional treatment improved BCVA in children with anisometropic amblyopia. They also pointed out that acupuncture may enhance activity in the occipital (inferior occipital gyrus) and temporal (fusiform and inferior temporal gyrus) lobes within the “WHAT” pathway, thereby alleviating visual impairment. Furthermore, emerging evidence suggests that acupuncture may benefit amblyopia management through multiple pathways, including repair of retinal ultrastructural damage, modulation of cortical neuronal excitability, and regulation of neurotrophic factor expression (20). Nevertheless, clinical studies on acupuncture for amblyopia are sparse and are predominantly small-sample, single-center trials, which curtail the precision and credibility of the clinical findings. Therefore, a more comprehensive and accurate evaluation of the clinical efficacy of acupuncture in amblyopia is imperative. In this study, we conducted a systematic meta-analysis of randomized controlled trials (RCTs) on acupuncture for amblyopia to assess the efficacy and safety of adding acupuncture to conventional therapies.

## 2 Methods

### 2.1 Literature search

We searched the PubMed, Embase, Cochrane Library, and Web of Science databases as well as the Chinese databases CNKI, Wanfang, VIP, and SinoMed for relevant literature published before December 31, 2024. The search fields were set to “Title/Abstract,” and the search strategy was defined as follows: [(Acupuncture OR Pharmacopuncture) AND (Amblyopia OR Amblyopias OR Lazy Eye OR Lazy Eyes)], with no other restrictions. The search strategies and results for each database are presented in Supplementary Table S1.

### 2.2 Inclusion and exclusion criteria

The inclusion criteria were as follows: (i) Study type: RCT. (ii) Study subjects: Conforming to the diagnostic criteria of the “Expert Consensus on Amblyopia Diagnosis (2011)” (21), “Definition, Classification, and Efficacy Evaluation Standards of Amblyopia” (22), or “Guidelines for the Diagnosis and Treatment of Amblyopia” (23). (iii) Intervention and comparison: The control group received conventional treatments such as refractive correction, patching therapy, penalization therapy, visual training, and medication, while the experimental group received acupuncture treatment based on the control group. (iv) Outcome indicators: The primary efficacy indicator was the clinical efficacy rate, defined as the proportion of patients who demonstrated significant improvement in vision following treatment (22). Specifically, efficacy cases were identified by comparing the uncorrected visual acuity before and after the intervention using a standardized vision chart. An improvement of at least two lines in visual acuity (measured as the number of optotype lines read correctly) in the treated eye compared to the baseline was considered indicative of effective treatment. Secondary efficacy indicators were BCVA, stereoacuity, and pattern visual evoked potential (P-VEP) P_100_ wave (latency and amplitude). The safety indicator was the total adverse reaction rate.

The exclusion criteria were as follows: (i) Animal experiments, retrospective studies, or case reports; (ii) repeatedly published studies; (iii) studies with missing baseline data; and (iv) studies with unavailable data.

### 2.3 Literature screening process

All retrieved articles were imported into EndNote X9. We initially excluded duplicate studies by reviewing the title, author, journal name, volume, issue number, and digital object identifier (DOI) of each article. Subsequently, the titles and abstracts of the articles were screened based on the inclusion criteria to exclude those irrelevant to the research topic. Finally, the full texts of the remaining studies were reviewed to determine the final list of included studies. Literature screening was independently conducted and cross-checked by KT and YY, and disagreements were resolved by PL.

### 2.4 Data extraction

The baseline characteristics and research data extracted from the included studies were systematically recorded in a Microsoft Excel 2024 spreadsheet. The recorded variables included the first author’s name, publication year, sample size, male ratio, average age, intervention, comparison, and treatment duration. Study data were defined as any measurement related to the outcome indicators. Baseline data and statistics were independently completed by KT and YY and cross-checked. In cases of disagreement, PL made the final decision.

### 2.5 Literature quality appraisal

The Risk of Bias 2 (RoB 2) tool was used to assess the risk of bias for the included studies. There were five bias domains in the RoB 2 tool: randomization process, deviations from intended interventions, missing outcome data, outcome measurement, selection of reported results. Based on the evaluation criteria defined in the Cochrane Handbook for Systematic Reviews of Interventions, the risk of bias for each domain was classified as low, high, or of some concern. This task was independently performed by KT and YY, who cross-checked each other’s work. In the event of any discrepancies, PL made the final judgment.

### 2.6 Statistical analysis

We utilized RevMan 5.3 (Cochrane Collaboration) to conduct meta-analyses, sensitivity analyses, and publication bias assessments following standardized systematic review procedures. For continuous variables, we calculated the mean difference (MD) with 95% confidence intervals (CIs), while for dichotomous variables, relative risk (RR) with 95% CIs was used as an effect size measure. The choice between the fixed-and random-effects models was based on the degree of heterogeneity assessed using the I^2^ statistic. Specifically, a fixed-effects model was applied when I^2^ ≤ 50%, indicating low heterogeneity. Conversely, when I^2^ was > 50%, indicating substantial heterogeneity, a random-effects model was selected to account for variability among the studies. All analyses were conducted using a significance threshold of *P* < 0.05.

Leave-one-out sensitivity analysis was performed to evaluate the robustness of the results. This involved sequentially excluding each individual study, re-running the meta-analysis each time, and examining the changes in the pooled effect size. If the overall results remained consistent without significant fluctuations, the findings were considered robust and unlikely to be driven by a single study.

Funnel plots were generated to visually inspect for publication bias. The symmetrical distribution of points on both sides of the funnel indicated the absence of publication bias, whereas asymmetry suggested potential bias. Egger’s test was also considered when enough studies (typically ≥ 10) were available to provide a more quantitative assessment, with *P* < 0.05 indicating possible bias.

### 2.7 Quality of evidence

The Grading of Recommendations Assessment, Development, and Evaluation (GRADE) approach was used to assess the quality of evidence of the outcomes. This involved evaluating the certainty of evidence based on domains such as the risk of bias, inconsistency, indirectness, imprecision, and publication bias. The overall quality of the evidence was rated as high, moderate, low, or very low, facilitating a transparent interpretation of the findings. This detailed methodological framework ensures a rigorous and transparent synthesis of the evidence, aligned with best practices in systematic reviews and meta-analyses.

## 3 Results

### 3.1 Literature screening

**FIGURE 1:**
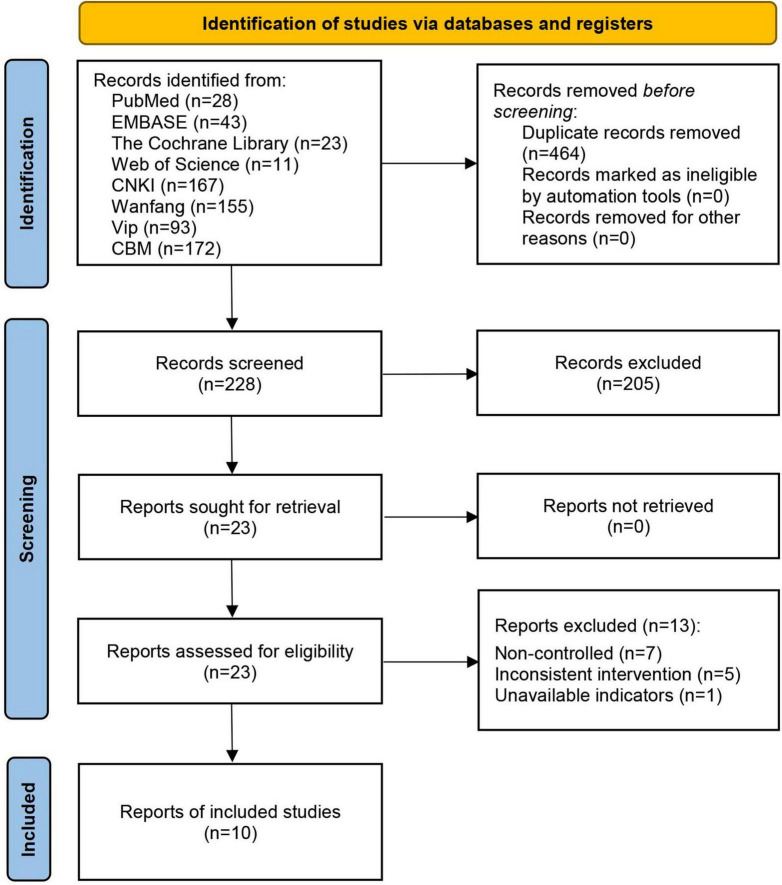
Literature screening flowchart.

A total of 692 articles were obtained from the eight databases. Among them, 464 articles were excluded because of duplication, and 205 articles were excluded because of irrelevant themes. During the full-text review, 13 articles were excluded for failing to meet the inclusion criteria, including 7 non-controlled trials, 5 studies with intervention deviations, and 1 study with unavailable metrics. Ultimately, 10 articles (24–33) were included in this meta-analysis, as shown in Figure 1.

### 3.2 Basic characteristics of the included studies

These studies included 918 amblyopic eyes, and their publication period ranged from 2010 to 2024. The average male ratio was 50.3%, the average age was 7.3 years, and the average treatment duration was 16 weeks. Specifically, nine studies (24–32) adopted refractive correction; eight (24–27, 29–32) used patching therapy; three (25, 29, 31) employed comprehensive treatment composed of red flash, grating, and visual stimulation; one used an amblyopia therapy device (24); one used pleoptic therapy (27); one adopted medium frequency electrotherapy (32); and one used visual training (33). See Table 1.

**TABLE 1:** Basic characteristics of the included studies.

Study	Sample size	Male/%	Age/ years	Intervention	Comparison	Course of treatment/ weeks
Ge and Liu (24)	86/80	47.8	/	Acupuncture, refractive correction, patching, and amblyopia therapy device	Refractive correction, patching, and amblyopia therapy device	48
Jia et al. (25)	39/39	/	9.0	Acupuncture, refractive correction, patching, red flash, grating, and visual stimulation	Refractive correction, patching, red flash, grating, and visual stimulation	4
Jin and Qiu (26)	66/64	52.3	6.6	Acupuncture, refractive correction, patching, pleoptic therapy, and Cambridge stimulator	Refractive correction, patching, pleoptic therapy, and Cambridge stimulator	24
Jin (27)	30/30	48.3	8.3	Acupuncture, refractive correction, patching, and pleoptic therapy	Refractive correction, patching, and pleoptic therapy	4
Liu (28)	50/44	51.1	5.4	Acupuncture and refractive correction	Refractive correction	12
Ma et al. (29)	36/38	52.7	8.6	Acupuncture, refractive correction, patching, red flash, grating, and visual stimulation	Refractive correction, patching, red flash, grating, and visual stimulation	4
Wang et al. (30)	30/30	/	/	Acupuncture, refractive correction, and patching	Refractive correction and patching	12
Ye et al. (31)	38/38	52.6	8.6	Acupuncture, refractive correction, patching, red flash, grating, and visual stimulation	Refractive correction, patching, red flash, grating, and visual stimulation	4
Zhao (32)	36/32	44.1	6.3	Acupuncture, refractive correction, patching, and medium frequency electrotherapy	Refractive correction, patching, and medium frequency electrotherapy	24
Zheng (33)	57/55	53.3	5.7	Acupuncture and visual training	Visual training	24

### 3.3 Literature quality appraisal

**FIGURE 2:**
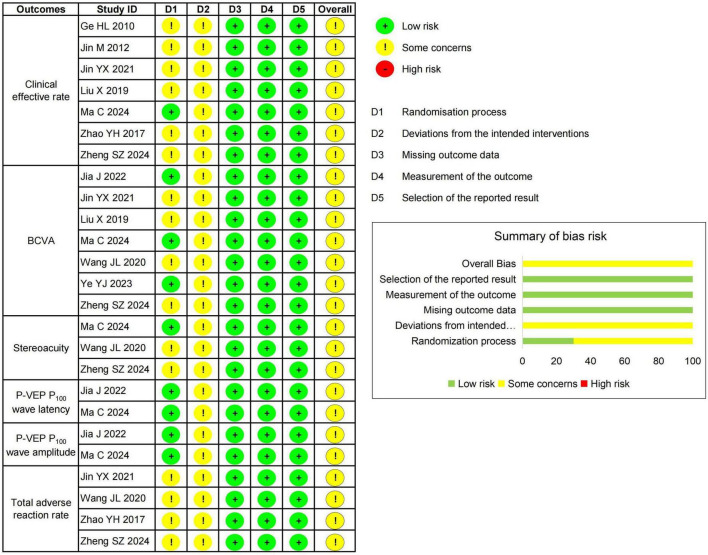
Risk of bias assessment.

Regarding the randomization process, three studies (25, 29, 31) were assessed as having a low risk of bias owing to their clear reporting of randomization methods (random number tables) and allocation concealment. In contrast, the other seven studies (24, 26–28, 30, 32, 33) had some concerns because they did not report details on random group assignment or allocation concealment. Concerning deviations from intended interventions, all 10 studies (24–33) were rated as having some concerns because of the lack of reported details on participant blinding. However, all participants strictly adhered to their assigned interventions throughout the trials, with no request for group switching or crossover interventions. With respect to missing outcome data, all studies adequately addressed this issue, ensuring that all registered participants had complete datasets and reported no dropouts. In terms of outcome measurements, all studies (24–33) provided data on outcomes along with appropriate statistical analyses, leading to the assessment of a low risk of bias. Furthermore, all studies (24–33) were evaluated as having a low risk of bias in the selection of reported results, as they listed pre-specified outcome measures in the section “2. Methods” and fully reported the changes observed in all participants’ laboratory indicators in the results section. Ultimately, the overall risk of bias for the 10 studies (24–33) was determined to be of some concern, as shown in Figure 2.

### 3.4 Meta-analysis

#### 3.4.1 Clinical efficacy rate

**FIGURE 3:**
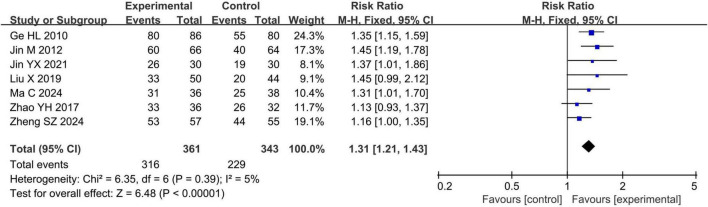
Forest plots of meta-analyses of the clinical efficacy rate.

Seven studies (24, 26–29, 32, 33) involving 704 eyes reported the clinical efficacy rate. The meta-analysis indicated that the clinical efficacy rate of the acupuncture combined treatment group was significantly higher than that of the conventional treatment group (RR = 1.31, 95% CI 1.21 to 1.43, *P* < 0.00001, I^2^ = 5%), as shown in Figure 3.

#### 3.4.2 BCVA

**FIGURE 4:**
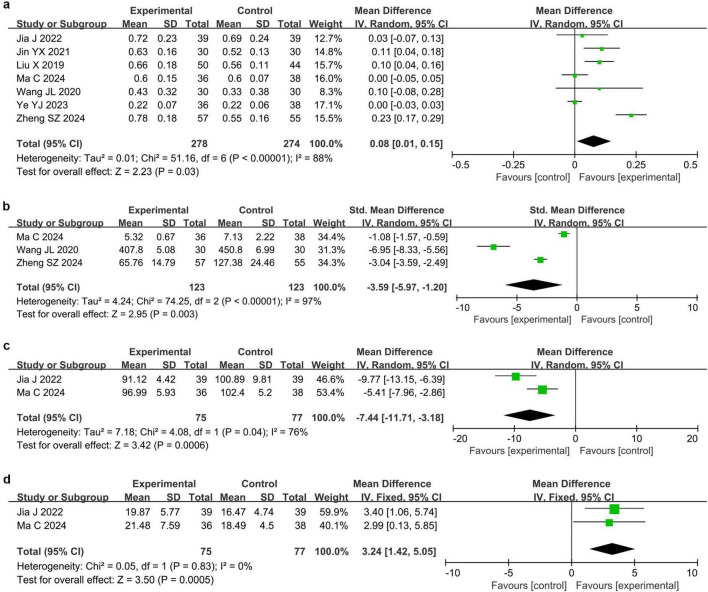
Forest plots of meta-analyses of secondary efficacy endpoints. **(a)** Best-corrected visual acuity (BCVA), **(b)** stereoacuity, **(c)** P-VEP P_100_ wave latency, and **(d)** Pattern visual evoked potential (P-VEP) P_100_ wave amplitude.

Seven studies (25, 27–31, 33) reported BCVA in 552 eyes. The meta-analysis revealed that BCVA was significantly higher in the acupuncture combined treatment group than in the conventional treatment group (MD = 0.08, 95% CI 0.01 to 0.15, *P* = 0.03, I^2^ = 88%), as shown in Figure 4a.

#### 3.4.3 Stereoacuity

Three studies (29, 30, 33) involving 246 eyes reported the stereoacuity. The meta-analysis demonstrated that the stereoacuity was significantly lower in the acupuncture combined treatment group than in the conventional treatment group (SMD = −3.59, 95% CI −5.97 to −1.20, *P* = 0.003, I^2^ = 97%), as presented in Figure 4b.

#### 3.4.4 P-VEP P_100_ wave latency and amplitude

Two studies (25, 29) reported changes in the latency and amplitude of the P-VEP P_100_ wave in 152 eyes. Meta-analysis showed that the latency of P-VEP P_100_ wave in the acupuncture combined treatment group was significantly shorter than that in the conventional treatment group (MD = −7.44, 95% CI −11.71 to −3.18, *P* = 0.0006, I^2^ = 76%), while the amplitude of P-VEP P_100_ wave was significantly higher than that in the conventional treatment group (MD = 3.24, 95% CI 1.42 to 5.05, *P* = 0.0005, I^2^ = 0%), as shown in Figures 4c, d.

#### 3.4.5 Adverse reaction rate

**FIGURE 5:**
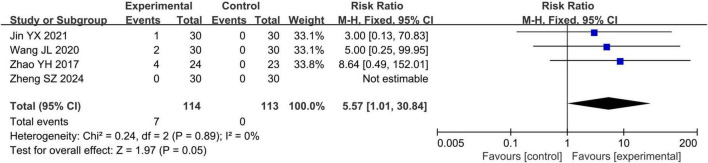
Forest plots of meta-analyses of the adverse reaction rate.

Four studies (27, 30, 32, 33) reported adverse reaction rate in 227 eyes. The meta-analysis indicated that the adverse reaction rate in the acupuncture combined treatment group was higher than those in the conventional treatment group (RR = 5.57, 95% CI 1.01 to 30.84, *P* = 0.05, I^2^ = 0%), as shown in Figure 5.

### 3.5 Sensitivity analysis

Sensitivity analysis revealed that the results for clinical efficacy rate and adverse reaction rate were robust, whereas those for BCVA and stereoacuity were not. Specifically, after excluding the study by Jin (27) (MD 0.07, 95% CI −0.00 to 0.15, *P* = 0.07) or Liu (28) (MD 0.08, 95% CI −0.01 to 0.16, *P* = 0.07), BCVA no longer had significance; after excluding the study by Zheng (33), stereoacuity no longer had significance (SMD = −3.98, 95% CI −9.72 to 1.77, *P* = 0.18). Additionally, because the latency and amplitude of P-VEP P_100_ wave included only two studies, no sensitivity analysis was conducted.

### 3.6 Publication bias

**FIGURE 6:**
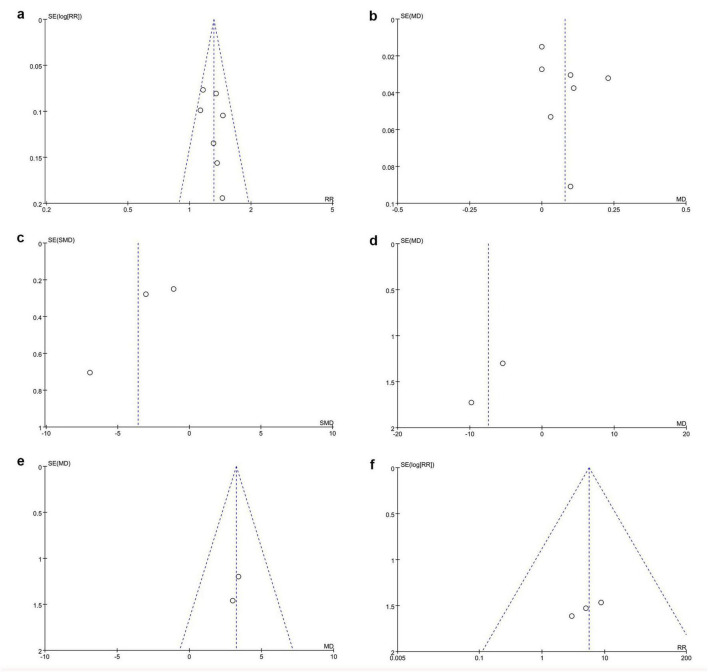
Funnel plots of publication bias. **(a)** Clinical efficacy rate, **(b)** BCVA, **(c)** stereoacuity, **(d)** P-VEP P_100_ wave latency, **(e)** P-VEP P_100_ wave amplitude, and **(f)** adverse reaction rate.

The funnel plots of the clinical efficacy rate, the latency of P-VEP P_100_ wave, the amplitude of P-VEP P_100_ wave, and the adverse reaction rate showed that the scattered points on both sides were roughly symmetrical, suggesting no publication bias. In contrast, the funnel plots of BCVA and stereoacuity exhibited asymmetrical scatter distributions, indicating the potential presence of publication bias (Figure 6). Additionally, as fewer than 10 studies were included for each outcome, Egger’s regression analysis was not performed.

### 3.7 Evidence quality

The quality of evidence for the clinical efficacy rate, the amplitude of P-VEP P_100_ wave, and the adverse reaction rate was low, while the quality of evidence for BCVA, stereoacuity, and the latency of P-VEP P_100_ wave was very low, as shown in Table 2.

**TABLE 2:** Quality of evidence.

Outcome	Risk of bias	Inconsistency	Indirectness	Imprecision	Publication bias	Quality of evidence
Clinical efficacy rate	Serious	None	None	Serious	None	Low
BCVA	Serious	Very serious	None	Serious	Serious	Very Low
Stereoacuity	Serious	Very serious	None	Serious	Serious	Very Low
P-VEP P_100_ wave latency	Serious	Very serious	None	Serious	None	Very Low
P-VEP P_100_ wave amplitude	Serious	None	None	Serious	None	Low
Adverse reaction rates	Serious	None	None	Serious	None	Low

## 4 Discussion

### 4.1 Research background and findings

Amblyopia, a common disorder in pediatric visual development, is the leading cause of visual impairment in children. When conventional treatment yield poor results (34), acupuncture has emerged as a highly regarded complementary treatment (17, 29). However, there is currently a lack of systematic reviews and meta-analyses evaluating the efficacy and safety of acupuncture for the treatment of amblyopia, and the specific value of acupuncture remains unclear. Therefore, it is necessary to synthesize and supplement evidence in this field using evidence-based approaches (35, 36). To our knowledge, this is the first meta-analysis to assess the efficacy and safety of acupuncture in amblyopia management. Our findings indicate that compared with the conventional treatment group, the acupuncture combined treatment group had a higher clinical efficacy rate, better BCVA, and higher amplitude of the P_100_ wave of P-VEP, while showing lower stereoacuity and shorter latency of the P-VEP P_100_ wave. However, the acupuncture combined treatment group may increase potential adverse events.

### 4.2 Efficacy of acupuncture in amblyopia

The clinical efficacy rate is a key indicator for evaluating the treatment effect of amblyopia and refers to the proportion of patients whose visual acuity has improved by two lines or more (22). Our study demonstrates that acupuncture, when combined with conventional treatments, significantly enhances the clinical efficacy rate in treating amblyopia. Previously, multiple clinical studies have suggested that the combination of acupuncture and conventional treatment methods leads to benefits in the clinical efficacy rate in amblyopia patients, which is consistent with the results of our study (27–29, 33, 37). Animal experiments have revealed the potential mechanisms of acupuncture in the treatment of amblyopia. These mechanisms include repairing neuronal structures, promoting visual electrophysiological functions, counteracting visual environmental disturbances, reversing the spontaneous firing frequency of the optic nerve, and transmission effects between central and target organs (38). Therefore, acupuncture can regulate the visual conduction pathway through multiple routes, thereby promoting the recovery of visual function (39). In conclusion, the relative benefit of the clinically efficacy rate provides strong evidence for acupuncture treatment of amblyopia.

Best-corrected visual acuity refers to the highest visual acuity that a patient can achieve after wearing corrective glasses or contact lenses. It is of vital importance in evaluating the efficacy of amblyopia treatment. Our meta-analysis showed that the improvement in BCVA was more significant in the acupuncture combined treatment group than in the conventional treatment group. In previous clinical studies, Ma et al. (29) and Jia et al. (25) reported that acupuncture significantly improved the BCVA in anisometropic amblyopia patients, supporting our findings. Improvements in the BCVA are of great significance in patients with amblyopia. On one hand, the improvement in BCVA enables patients to perceive the world more clearly, enhancing their quality of life. On the other hand, it can prevent strabismus, promote the development of the visual system, and ensure long-term stability of vision (40). Cui et al. (18) utilized resting-state functional magnetic resonance imaging (rs-fMRI) to demonstrate that acupuncture augments neuronal activity in the inferotemporal cortex within the ventral “what” pathway, optimizing visual information processing and alleviating shape/color perception deficits in amblyopia. This further supports the value of acupuncture in the treatment of amblyopia and provides evidence for its clinical application. However, the sensitivity analysis showed that the results of BCVA were not robust, indicating that the aforementioned benefits need to be further evaluated in future clinical trials.

Stereoscopic vision, the ability of the visual system to perceive the distance, depth, and convexity or concavity of three-dimensional objects, is of great significance for an individual’s spatial cognition and daily activities. As a key indicator for quantifying the accuracy of this perception, the importance of stereoacuity is self-evident (41). This meta-analysis shows that acupuncture combined with conventional treatment effectively reduces stereoacuity. Previous clinical studies conducted by Wang et al. (30), Ma et al. (29), and Zheng (33) also reported that adding acupuncture to conventional treatments can significantly reduce stereoacuity, supporting our findings. This indicates that acupuncture improves performance in tasks requiring fine manipulation and depth perception in patients with amblyopia, thereby enhancing their quality of life and social participation (42). Stereoscopic vision reconstruction is closely related to amblyopia, and the more severe the amblyopia, the more difficult it becomes (43). Patients with mild and moderate amblyopia have a relatively easier establishment of ideal stereoscopic vision, whereas patients with severe amblyopia face greater challenges. When the visual acuity of the affected eye is < 0.6, perfect stereoscopic vision is difficult to achieve (26). Nevertheless, clinical trials by Wang et al. (30) indicated that acupuncture combined with conventional treatment significantly improved the stereoscopic vision of patients with severe or refractory amblyopia, further highlighting the potential of acupuncture in the treatment of amblyopia.

Pattern visual evoked potential is the electrical activity generated by the occipital visual cortex in response to flash or graphic stimuli from the retina. It is an important electrophysiological indicator of visual function in amblyopic patients (44). The P_100_ wave in P-VEP has special significance. Its latency represents the speed of information transmission from retinal ganglion cells to the visual cortex, and the amplitude of the P_100_ wave reflects the excitability of visual cortex neurons. The higher the amplitude, the stronger the excitability of the visual cortex neurons. Our meta-analysis shows that acupuncture significantly shortens the latency of P-VEP P_100_ wave and increases the amplitude of P-VEP P_100_ wave. Ma et al. (29) also reported that after treatment, in the acupuncture group, the latency of P-VEP P_100_ wave was shorter and the amplitude of P-VEP P_100_ wave was higher, which is consistent with our findings. Subsequent mechanism studies have shown that acupuncture enhances visual nerve transmission through brain regions related to the limbic system, frontal lobe, and parietal lobe (25). Additionally, acupuncture enhances the bioelectric activity of the visual nerve cells to improve the efficiency of visual nerve conduction, thereby effectively regulating the inhibition and retardation of visual responses caused by visual deprivation, particularly during the sensitive period of visual development (45). The benefits of acupuncture in the P-VEP P_100_ wave reflect its positive impact on visual development.

### 4.3 Safety of acupuncture in amblyopia

In this meta-analysis, the adverse event rate in the acupuncture combined treatment group was 6.1% (7/114), whereas that in the conventional treatment group was 0% (0/113). The adverse reaction rate in the acupuncture combined treatment group was higher than those in the conventional treatment group. Nevertheless, among the included studies, seven adverse events were potentially associated with acupuncture. Specifically, four patients refused further acupuncture because of fear, one patient experienced dizziness due to nervousness during the procedure; another patient felt dizziness when being treated on an empty stomach, and one patient had local bleeding after acupuncture at the periorbital acupoints. Children, who are the major population of patients with amblyopia, are highly likely to develop negative emotions such as fear and anxiety when undergoing acupuncture, especially when periorbital acupoints are targeted (46, 47). Thus, before performing acupuncture operations, practitioners need to communicate fully with the patients and their families and do a good job at providing psychological comfort for the children (48). This will effectively reduce the occurrence of such adverse events. Moreover, local bleeding and bruising at periorbital acupoints are adverse events of acupuncture (49). To effectively reduce such adverse events, practitioners must have a comprehensive understanding of the anatomical structure of the eyes and receive professional training (50). In conclusion, although the meta-analysis indicates that acupuncture may increase the risk of potential adverse events, these events are generally mild and related to the operation. Therefore, caution should be exercised during needle insertion, and the patient’s condition should be closely monitored to minimize the occurrence of acupuncture-related adverse events during the operation.

### 4.4 Limitations and prospects

Our study had certain limitations. First, the number of included studies and sample size were relatively small, which limited the precision of the results. Second, the included studies had methodological biases, including randomization, blinding, and masking. Third, all participants in the included studies were Chinese, restricting the generalizability of the findings to a limited population. Fourth, the acupoints, depth, and retention time of acupuncture may be potential sources of clinical heterogeneity; however, owing to the limited research basis, further analysis cannot be conducted. In the future, we expect to conduct high-quality, large-sample, multicenter RCTs to comprehensively and accurately evaluate the efficacy and safety of acupuncture treatment for amblyopia. Furthermore, it is necessary to further explore the acupoints, needling depth, and needle retention time in clinical trials to formulate a complete acupuncture treatment protocol for amblyopia.

## 5 Conclusion

Compared to conventional treatment, acupuncture combined with conventional treatment improved the visual status of patients with amblyopia, although it may increase the risk of adverse events. Considering that these adverse events are mild, acupuncture still has the potential to serve as a complementary treatment for amblyopia. It offers an evidence-based option for clinical practice, potentially improving patient outcomes and contributing to amblyopia management. However, owing to the limited sample size and methodological quality, these findings need to be further evaluated in multicenter, high-quality clinical studies.

## Data Availability

The original contributions presented in this study are included in this article/Supplementary material, further inquiries can be directed to the corresponding authors.
